# Efficacy of anti-VEGF in the treatment of choroidal neovascular membrane secondary to pattern dystrophy simulating fundus flavimaculatus

**DOI:** 10.3205/oc000110

**Published:** 2019-06-18

**Authors:** Purna Nangia, Dhaivat Shah, Kumar Saurabh, Rupak Roy

**Affiliations:** 1 Department of Vitreo Retina, Aditya Birla Sankara Nethralaya, Kolkata, India

## Abstract

Pattern dystrophies are a group of inherited disorders of the retinal pigment epithelium. A 44-year-old female came with loss of vision in her right eye. The fundus of both eyes showed flecks in the posterior pole with a CNVM in the right eye. FFA and SD OCT confirmed the presence of CNVM. The patient underwent treatment with anti-VEGF injection. Post treatment, the vision improved with a reduction in subretinal fluid along with a scarring CNVM. To conclude, we report an extremely rare case of PDSFF associated CNVM and its favourable response to anti-VEGF injection.

## Introduction

Pattern dystrophies (PD) are a group of inherited disorders of the retinal pigment epithelium (RPE) characterized by deposits over the posterior pole [1]. These disorders are primarily benign, but can have secondary pathogenic manifestations. PDs are divided into 5 types based on the pattern of pigment distribution: adult-onset foveo macular vitelliform dystrophy (AOFVD), butterfly-shaped pigment dystrophy, reticular dystrophy of the retinal pigment epithelium, pattern dystrophy simulating fundus flavimaculatus (PDSFF), and fundus pulverulentus [2]. PDSFF is characterized by yellowish flecks over the posterior pole and extending beyond the arcades [3]. PDs usually have a good visual prognosis till later life, but can be rarely associated with other complications like choroidal neovascular membrane (CNVM). CNVM is reported with butterfly, reticular, and foveomacular PD, but there is only a single case report of CNVM associated with PDSFF in published literature [4]. Here we present an extremely rare case of PDSFF with CNVM and its excellent response to anti-VEGF treatment. 

## Case description

**Figure 1:**
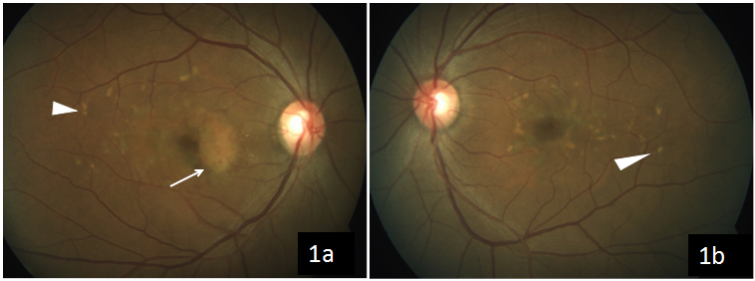
Color fundus photograph of the right eye (1a) showing multiple yellowish flecks over the posterior pole with a pale yellow confluent lesion with irregular borders nasal to the fovea with a speck of hemorrhage over it suggestive of CNVM; left eye (1b) showing multiple yellow flecks over the posterior pole.

**Figure 2:**
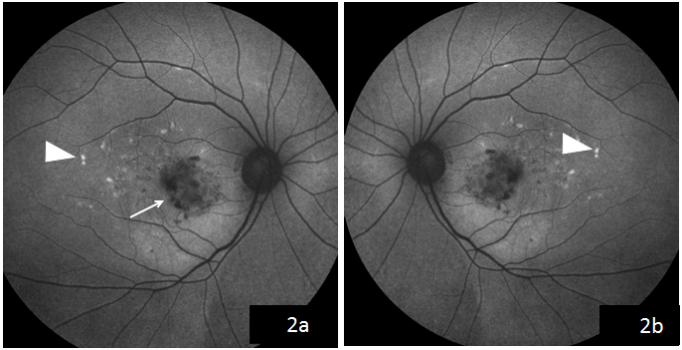
Blue autofluorescence image of the right eye (2a) showing multiple hyperautofluorescent lesions corresponding to flecks and a hypoautofluorescent lesion corresponding to the CNVM in color fundus; left eye (2b) showing multiple hyperautofluorescent lesions corresponding to flecks.

**Figure 3:**
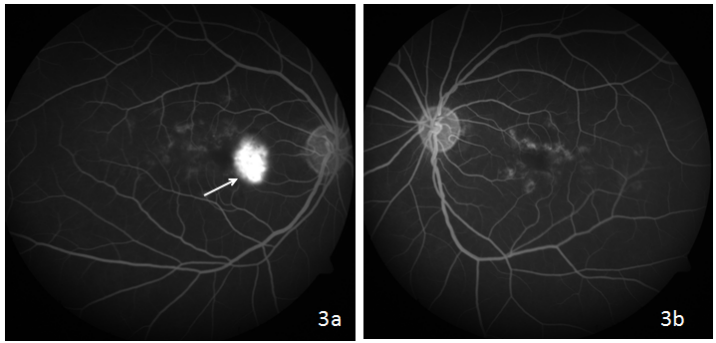
Fundus fluorescence angiography of the right eye (3a) showing an area of increasing hyperfluorescence in late phase suggestive of classical CNVM leakage with small areas of hyperfluorescence staining corresponding to flecks; left eye (3b) showing small areas of hyperfluorescence staining corresponding to flecks.

**Figure 4:**
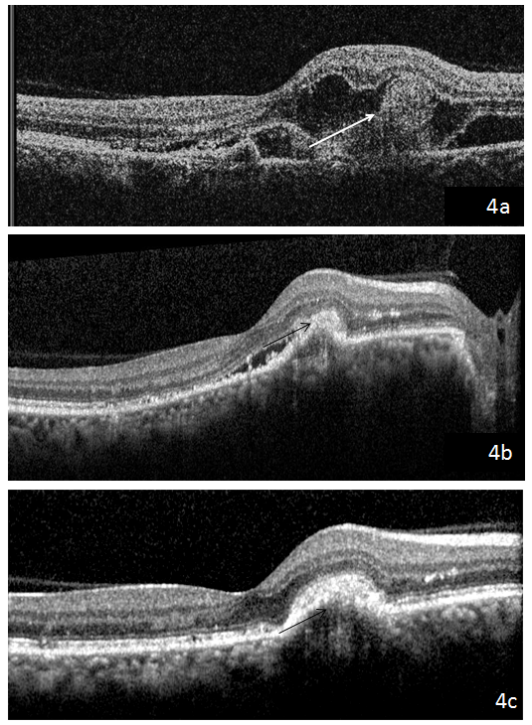
SD OCT of the right eye pre treatment (4a) showing a juxtafoveal CNVM with surrounding subretinal fluid; the right eye post anti-VEGF treatment (4b) shows minimal subretinal fluid with regressing CNVM and adjacent scarring. The right eye post second anti-VEGF injection (4c) shows completely regressed subretinal fluid.

A 44-year-old female came to us with the complaint of gradual visual loss and metamorphopsia in the right eye since 6 months. At presentation, she had a BCVA of 20/40 N6 in the right and 20/30 N6 in the left eye, respectively. Anterior segment examination was normal. On fundus examination, the right eye showed multiple yellowish flecks over the posterior pole and a pale yellow confluent lesion with irregular borders nasal to the fovea with a speck of hemorrhage over it, while the left eye showed multiple yellow flecks over the posterior pole (Figure 1 (Fig. 1)). Blue autofluorescence image of both eyes showed multiple hyperautofluorescent lesions corresponding to flecks and the right eye showed a hypoautofluorescent lesion corresponding to the CNVM (Figure 2 (Fig. 2)). Fundus fluorescein angiography (FFA) of the right eye (Figure 3a (Fig. 3)) showed an area of increasing hyperfluorescence in late phase suggestive of classic CNVM with small areas of hyperfluorescent staining corresponding to flecks; the left eye (Figure 3b (Fig. 3)) showed small areas of hyperfluorescence staining corresponding to flecks. Spectral domain optical coherence tomography (SD OCT) of the right eye pre treatment (Figure 4a (Fig. 4)) showed a juxta foveal CNVM with surrounding subretinal fluid. A full field electroretinogram was done which was found to be normal. In view of these findings, a diagnosis of PDSFF with classic CNVM was made. The patient was advised anti-VEGF injection. Subsequently, the patient went ahead with injections of Ranibizumab in her right eye. The patient came for a follow-up visit after one month. At this visit, her BCVA improved to 20/30 N6 with a significant decrease in her metamorphopsia. A repeat SD OCT was done (Figure 4b (Fig. 4)) which showed minimal subretinal fluid with regressing CNVM and adjacent scarring. The patient was advised further anti-VEGF injections and the patient went ahead with injection of Ranibizumab in her right eye. Repeat OCT was done on follow-up visit after a month which showed a complete regression of the subretinal fluid and her vision was maintained (Figure 4c (Fig. 4)). 

## Discussion

PDs are a group of retinal disorders which predominantly affect the RPE. The visual acuity remains stable in most of these conditions, until and unless complicated by a secondary pathology like CNVM [1]. Sparse information regarding the visual prognosis for PD cases complicated with CNVM is available in the published literature. 

Battaglia Parodi et al. studied a series of 9 patients with PD and CNVM. Their series included four cases of reticular dystrophy, one case of butterfly-shaped dystrophy, three cases of AOFVD, and one case of PDSFF. They treated all the patients with PDT with subsequent scarring of CNVM [4].

Management of CNVM has undergone a paradigm shift due to the introduction of anti-VEGF agents in its treatment. Excellent results were obtained in treatment of CNVM secondary to ARMD and other etiologies like myopia with anti-VEGF agents [1], [5].

Little information is available regarding the efficacy of anti-VEGF in the treatment of pattern dystrophy associated CNVM. Battaglia Parodi et al. studied a series of 12 patients with PD and CNVM who were treated with a course of intravitreal bevacizumab for a period of more than 2 years and documented a good anatomical and functional outcome [5]. Gallego-Pinazo et al. published a series of 6 females diagnosed with AOFVD related CNVM treated with ranibizumab intravitreal injections. This treatment was found to be effective in increasing visual acuity over a short term [6]. Empeslidis et al. documented the effectiveness of intravitreal ranibizumab in the treatment of patients with choroidal neovascularization due to butterfly-shaped pattern dystrophy of the macula [1].

CNVM in PDSFF is extremely rare. There exists only one case in published literature described by Battaglia Parodi et al. where PDSFF with CNVM was treated with PDT [4]. The baseline BCVA was 20/100. The patient underwent 2 PDT sessions and the CNVM underwent fibrosis. The BCVA at final follow-up was 20/25. Our case had PDSFF with classic CNVM. Fundus flavimaculatus/Stargardt disease was considered as a differential diagnosis in our case. However, a normal full field electroretinogram, absence of choroidal silence in FFA along with preservation of good vision, and characteristic triradiate flecks over the posterior pole clinched the diagnosis of PDSFF. Genetic analysis of this case would have been a necessary addendum to above clinical findings, lack of which is a limitation of our report. No case of PDSFF with CNVM treated by anti-VEGF injection has been described in published literature as of yet. Our case had a good resolution of subretinal fluid with an increase in visual acuity post anti-VEGF injection.

To conclude, we report an extremely rare case of PDSFF associated CNVM and its favourable response to anti-VEGF injection.

## Notes

### Competing interests

The authors declare that they have no competing interests.
